# Social distancing in advanced emergency medicine courses – can it work?

**DOI:** 10.3205/zma001418

**Published:** 2021-01-28

**Authors:** Robert Gintrowicz, Klemens Pawloy, Antje Degel

**Affiliations:** 1 Charité Universitätsmedizin Berlin, Prodekanat für Studium und Lehre, Berlin, Germany; 2 Charité Universitätsmedizin Berlin, Med. Klinik für Kardiologie, Campus Benjamin Franklin, Berlin, Germany

**Keywords:** emergency medicine, simulation, COVID-19, hygienic standards, blended learning

## Abstract

**Introduction: **The corona virus pandemic rendered most live education this spring term impossible. Many classes were converted into e-learning formats. But not all learning content and outcomes can readily be transferred into digital space.

**Project outline: **Emergency medicine teaching relies on hands-on simulation training. Therefore, we had to devise a catalogue of measures, that would enable us to offer simulation training for Advanced Life Support.

**Summary of work:** Strict hygienic rules including disinfection of hands, wearing personal protective gear at all times and disinfection of equipment were implemented. Group size and number of staff was reduced, introducing fixed student teams accompanied by the same teacher. Only large rooms with good ventilation were used. Under these conditions, we were allowed to carry out core Advanced Life Support simulations. Other content had to be transferred to online platforms.

**Discussion:** Heeding general hygiene advise and using personal protective gear, a central cluster of simulations was carried out. Students and staff adhered to rules without complaint. No infections within faculty or student body were reported.

**Conclusion: **It seems feasible to conduct core simulations under strict hygienic protocol.

## Zusammenfassung

**Einführung:** Die Coronavirus-Pandemie machte in diesem Sommersemester Präsenzunterricht praktisch unmöglich. Viele Veranstaltungen wurden in E-Learning-Formate umgewandelt. Aber nicht alle Lerninhalte und -ziele lassen sich ohne weiteres in den digitalen Raum übertragen.

**Projektskizze: **Der Unterricht in der Notfallmedizin stützt sich auf praktisches Simulationstraining. Deshalb mussten wir einen Maßnahmenkatalog erarbeiten, der es uns ermöglicht, Simulationstraining für Advanced Life Support anzubieten.

**Zusammenfassung der Arbeit: **Strenge Hygienevorschriften einschließlich der regelmäßigen Desinfektion der Hände, des durchgehenden Tragens persönlicher Schutzausrüstung und der Desinfektion der Ausrüstung wurden umgesetzt. Die Gruppengröße und die Anzahl der Mitarbeiter wurden reduziert. Feste Studierendenteams in Begleitung desselben Dozierenden wurden eingeführt. Es wurden nur noch große Räume mit guter Belüftung verwendet. Unter diesen Bedingungen durften wir die Schlüsselsimulationen zum Advanced Life Support durchführen. Andere Inhalte mussten auf Online-Plattformen übertragen werden.

**Diskussion: **Unter Beachtung der allgemeinen Hygieneberatung und unter Verwendung persönlicher Schutzausrüstung wurde ein zentrales Cluster von Simulationen durchgeführt. Die Studierenden und das Personal hielten sich problemlos an die Regeln. Es wurden keine Infektionen innerhalb der Fakultät oder der Studentenschaft gemeldet.

**Schlussfolgerung: **Es scheint vertretbar zu sein, Kernsimulationen unter streng hygienischen Bedingungen durchzuführen.

## Introduction

The beginning of spring semester 2020 was postponed due to corona virus pandemic and teaching thereafter was mostly converted into distance formats [1]. Some formats though cannot be offered in digital forms with the same qualitative outcomes. Emergency medicine are usually taught in simulations [2], [3], [4], [5]. The combination of technical and non-technical skill training, teamwork, leadership and crew resource management is not easily compensated with e-learning formats, as non-simulation teaching showed inferiority regarding learner satisfaction and process skill [6]. The European Resuscitation Council (ERC) recommends on-site simulations with subsequent debriefing for advanced life support (ALS) psychomotor and non-technical skill training [7]. Cognitive learning of background information can and has been successfully transferred into digital learning by the ERC.

## Project outline

The 10^th^ semester emergency medicine course usually comprises 16 simulations between 90 and 180 minutes of duration covering internal medicine, traumatic, neurologic pediatric and obstetric emergencies for 319 medical students (see table 1 (Tab. 1)). Six of these simulations are taught interdisciplinary (anesthesiology, internal medicine, traumatology), one is taught with the help of two simulation patients, two are taught in a tandem of a clinical teacher and a peer student teacher and seven are taught by a single clinical teacher. In these simulations nine students are divided into subgroups that have to work through emergency scenarios as a team. This warrants close personal contact and heavy physical work in the case of cardiac compressions. This did not comply with social distancing rules as decreed by the Federal Ministry of Health (Bundesministerium für Gesundheit) [8]. We therefore had to find alternatives for the execution of the simulations focusing on psychomotor and non-technical skills to be allowed to carry these out on-site as well as develop digital theoretical background support.

## Summary of work

The minimum standard of emergency medicine proficiency was set according to advanced life support outcomes as stated by the European Resuscitation Council. A certificate in ALS is a basic requirement for medical practice in many European health care systems. Therefore, nine simulations encompassing these contents had to be adapted to the required hygienic standards [9]. In Basic Life Support (BLS) compression-only-CPR was taught, applying the pre-pandemic algorithm for educational purposes (last course prior to graduation). The modified algorithm for COVID-19 for laypeople (ERC) [10] and medical professionals (American Heart Association) [11] was offered online. The problems concerning the emergency treatment of patients with infectious diseases or unknown status were introduced in this session and reiterated in all of the following. The other eight simulation courses focusing on trauma, obstetrics or ethics for example were transferred into online lectures and seminars including video cases. Semester evaluation and course evaluation were offered.

In cooperation with the Institute of Hygiene and the COVID-19 task force we established the following rules:

1. Small group learning (no more than nine) with only one single teacher (no contact between different departments) through all sessions.
2. Admission to class for students and teachers only after hand disinfection and wearing a face mask for the entire duration of the session.
3. Fixed three-student-subgroups were defined for the duration of the entire course (all nine simulations), within these groups individuals were exempt from social distancing (wearing masks and gloves) enabling resuscitation efforts in simulations. In between subgroups, a minimum distance of 2 meters was observed.
4. Distance between students and teacher was over 2 meters at all times.
5. Only large training rooms (35-45 m^2^) with many windows for air circulation were used. Some sessions (if applicable to content, eg. Basic Life Support) were held outside in the patio. Windows were held open during the entire session (warm temperatures allowed this proceeding).
6. Manikins and equipment were disinfected according to guidelines between scenarios.
7. Rooms were disinfected according to guidelines between sessions.
8. Mouth-to-mouth ventilations in BLS classes were not allowed.
9. These actions were communicated via email to all participants, posters were installed in all rooms and the halls.
10. Two simulation center employees surveilled adherence to measures.

Students and teachers readily complied with said hygienic measures. Motivation to perform was high, participants were happy to be able to train ALS. Continuously wearing face masks and gloves, disinfecting manikin and equipment did not deter from participation. All students were tested once prior and twice after the simulation classes. No SARS-CoV-2 infections were found among students from ALS classes.

The transfer of scenarios into the digital space was challenging given the short amount of time available. Therefore some topics, such as pediatrics, used professional video cases, whereas for others cases were developed locally.

Semester evaluation was returned by 22 of 319 students, course evaluation was completed by 156 of 319 students. Both evaluations showed that many students were overall glad they were able to partake in the course albeit the pandemic. 86% reviewed the emergency course as “excellent” or “good”, this is slightly less than in prior semesters (averaging around 90-95% for this evaluation). Criticism pertaining to the transfer of on-site simulations into online learning, especially those without online seminar or video lectures, was recorded by 3 of the 22 students in semester evaluation, 29 of 156 students in the course evaluation.

## Discussion

We propose a catalogue of measures how live sessions involving team work in close physical proximity could be implemented under COVID-19 conditions. Of course, there is no absolute elimination of contagion risk [9]. Since none of our 319 students tested positive for SARS-CoV-2, adhering to hygienic standards, using face masks and gloves as well as working with fixed teams, seems to be a feasible alternative to be able to offer students much needed simulation training in emergency medicine. Current topics on hygienic considerations in emergency situations can be implemented into pre-existing cases. 

Transfer of sessions into online learning has to be carefully weighted, since emergency courses heavily rely on skill acquisition, opportunity to learn in application to simulation cases as well as non-technical skill training and debriefing [7]. Mere knowledge acquisition does not suffice. Therefore, new formats – probably blended – will be needed to satisfy these needs as well as pandemic restraints.

## Conclusion

Strict hygienic measures can be upheld during simulation classes and so enable the execution of emergency medicine training as shown via negative results of cohort testing.

## Competing interests

The authors declare that they have no competing interests. 

## Figures and Tables

**Table 1:**
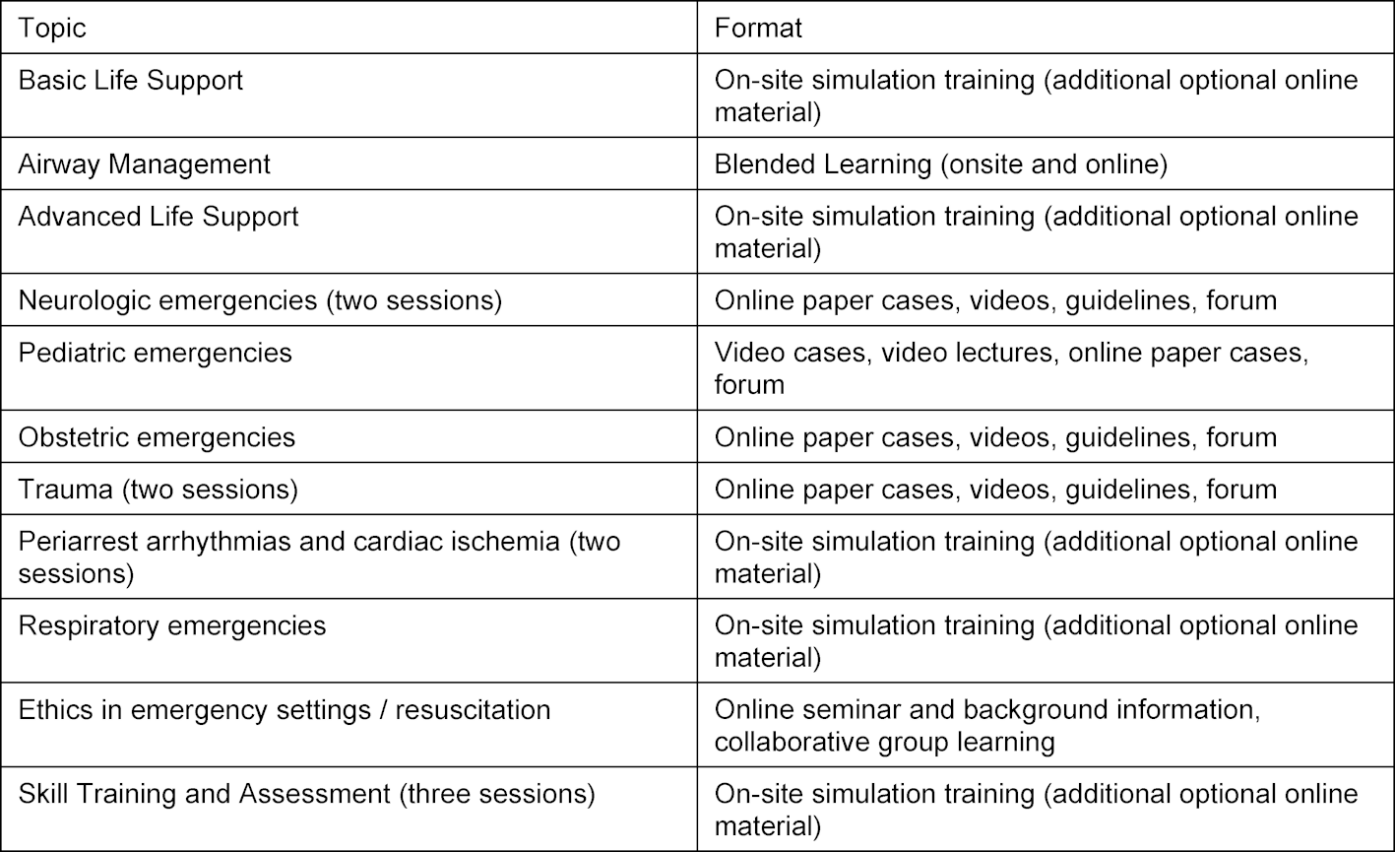
Emergency Medicine course – pandemic adaptation of courses
